# Diversity and Distribution of *Borrelia hermsii*

**DOI:** 10.3201/eid1303.060958

**Published:** 2007-03

**Authors:** Tom G. Schwan, Sandra J. Raffel, Merry E. Schrumpf, Stephen F. Porcella

**Affiliations:** * National Institute of Allergy and Infectious Diseases, Hamilton, Montana, USA

## Abstract

Borrelia hermsii is the most common cause of tickborne relapsing fever in North America. DNA sequences of the 16S–23S rDNA noncoding intergenic spacer (IGS) region were determined for 37 isolates of this spirochete. These sequences distinguished the 2 genomic groups of B. hermsii identified previously with other loci. Multiple IGS genotypes were identified among isolates from an island, which suggested that birds might play a role in dispersing these spirochetes in nature. In support of this theory, all stages of the tick vector Ornithodoros hermsi fed successfully on birds in the laboratory and advanced in their life cycle. B. hermsii produced a detectable spirochetemia in 1 chicken inoculated subcutaneously. Additional work is warranted to explore the role of birds as enzootic hosts for this relapsing fever spirochete.

## Abstract 2

Multilocus sequence analysis and laboratory experiments suggest that birds may play a role in maintaining and dispersing this pathogen.

Tickborne relapsing fever in humans in North America is most often caused by the spirochete Borrelia hermsii, which is transmitted by its argasid tick vector, Ornithodoros hermsi (*1*). The spirochete is endemic to the western United States and southern British Columbia in Canada (Figure 1) but restricted to higher elevations with coniferous forests where both the ticks and appropriate vertebrate hosts coexist (*1*). The most common exposure for humans occurs while they are sleeping in tick-infested cabins, where the nocturnal ticks seek their hosts and feed quickly within 15 to 90 minutes and then return to their refuge in the walls, floor, or attic.

**Figure 1:**
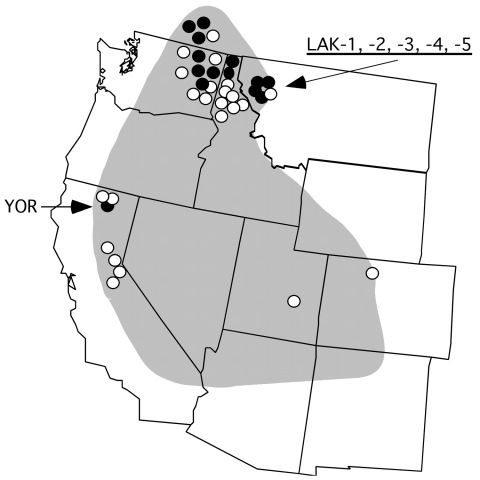
Western United States showing the approximate endemic range of tickborne relapsing fever associated with Ornithodoros hermsi and the localities of origin for the 37 Borrelia hermsii isolates included in this study. Genome group I (GGI) isolates are shown by open circle; GGII isolates are shown by filled circle. Localities of 6 isolates discussed in detail are indicated with arrows.

The specific association of this spirochete with O. hermsi led to the bacterium’s being named B. hermsii, to distinguish it from other species of relapsing fever spirochetes transmitted by other species of ticks in the western United States (*2*). The ability to propagate B. hermsii in pure culture (*3*) and the development of molecular techniques and databases to identify, type, and compare spirochetes were critical advances for the study of these bacteria. We are now able to characterize and better define these species and to elucidate the geographic distribution and role that O. hermsi and various vertebrate hosts play in maintaining B. hermsii in nature. These advances are countered, however, by the difficulty in finding infected O. hermsi ticks or rodents in the wild and the infrequent access to infected blood samples from patients when they are acutely ill and spirochetemic. Additionally, although B. hermsii is cultivatable, establishing these spirochetes in vitro from infected samples is not always successful.

Recently, we identified 2 genomic groups in B. hermsii by multilocus sequence typing of 31 isolates (*4*). Four loci were examined (16S rRNA, flaB, gyrB, and glpQ), which cumulatively totaled 5,197–5,203 bp per isolate. The 2 genomic groups of B. hermsii were also distinct from isolates of B. turicatae and B. parkeri, for which we undertook a similar analysis (*5*). Bunikis and co-workers recently typed relapsing fever spirochetes based on the intergenic spacer (IGS) region of noncoding DNA located between the 16S rRNA and ileT tRNA genes (*6*). In their report, 4 IGS types were identified among 9 isolates or DNA extracted from tissues infected with B. hermsii. Given that less effort is needed to type B. hermsii with only the IGS sequence than to several larger loci, we undertook an analysis of the IGS region in our isolates to determine its utility to define the 2 genomic groups in these spirochetes. Here we compare results obtained with the IGS locus to results obtained by multilocus sequence typing, which included recently acquired spirochetes from an outbreak of relapsing fever that were not examined previously. We use these data to discuss the geographic distribution of B. hermsii and explain how birds may help maintain and disperse these spirochetes in nature.

## Materials and Methods

The B. hermsii examined in this study originated from infected humans (n = 32), O. hermsi ticks (n = 4), and 1 chipmunk (Table 1). Isolates were established by first inoculating laboratory mice (Mus musculus) and then passing infected mouse blood into BSK-H medium with 12% rabbit serum (Sigma-Aldrich, Saint Louis, MO, USA) (*4*). Genomic DNA samples were prepared from pure cultures (*7*), and PCR amplification and DNA sequencing of 16S rRNA, flaB, gyrB, and glpQ were completed as described (*4*). The IGS sequences were determined by PCR amplification with primers IGS-F and IGS-R (*8*) and an initial heating at 96°C for 3 min, followed by 35 cycles with denaturation at 96°C for 30 s, annealing at 55°C for 30 s, and extension at 72°C for 2 min. After the 35th cycle, an additional extension was done at 72°C for 7 min. DNA sequences of the amplicons were determined with primers IGS-F, IGS-R, Fn, and Rn (*8*).

**Table 1:** *Borrelia hermsii* isolates examined, history, origin, and IGS sequence types, western North America*

Isolate*	Year	Source	Locality	IGS type†
GGI				
HS1	1968	Tick	Spokane Co., WA	1
CON	1960s	Human	Sierra Nevada Mtns, CA	1
FRO	1987	Human	Eastern WA	1
DAH	1991	Human	Spokane Co., WA	1
FRE	1996	Human	Pend Oreille Co., WA	1
MIL	1996	Human	Kootenai Co., ID	1
BRO	1996	Human	Kootenai Co., ID	1
CAR	1996	Human	Benewah Co.,ID	1
BAK	1997	Human	Okanogan Co., WA	1
BYM	1997	Human	Kootenai Co., ID	1
RAL	1997	Human	Siskiyou Co., CA	1
SIS	1998	Ticks	Siskiyou Co., CA	1
HAL	1998	Human	Kootenai Co., ID	1
GAR	2001	Human	Okanagan Valley, BC	1
LAK-4	2004	Human	Lake Co., MT	1
MAT	2004	Human	Bitterroot–Selway Mtns, ID	1
DOU	2005	Ticks‡	Douglas Co., WA	1
EST-7	1996	Chipmunk	Larimer Co., CO	2
ALL	1997	Human	Duchesne Co., UT	2
WAD	1998	Human	Placer Co., CA	2
SWA	1996	Human	Kootenai Co., ID	5
ELD	2005	Ticks‡	Eldorado Co., CA	6
MAN	1960s	Human	Sierra Nevada Mtns, CA	7
GGII				
YOR	1964	Human	Siskiyou Co., CA	8
REN	1992	Human	Okanogan Co., WA	8
OKA-1	1995	Human	Okanagan Valley, BC	8
OKA-2	1996	Human	Okanagan Valley, BC	8
OKA-3	1996	Human	Okanagan Valley, BC	8
GMC	1997	Human	Stevens Co., WA	8
CMC	1997	Human	Stevens Co., WA	8
LAK-3	2004	Human	Lake Co., MT	8
LAK-5	2004	Human	Lake Co., MT	8
LAK-1	2002	Human	Lake Co., MT	9
LAK-2	2002	Human	Lake Co., MT	9
SIL	2002	Human	Boundary Co., ID	9
HAN	1990	Human	Boundary Co., ID	9
RUM	1997	Human	Stevens Co., WA	10

*GGI, genomic group I; GGII, genomic group II; WA, Washington; Mtns, mountains; Co., county; ID, Idaho; CA, California, BC, British Columbia; MT, Montana; CO, Colorado; UT, Utah. †Each IGS sequence type is unique from the others and represented by the following isolate and GenBank accession nos.: type 1, DAH, DQ845746; type 2, ALL, DQ845747; type 5, SWA, DQ845746; type 6, ELD, DQ845745; type 7, MAN, DQ845748; type 8, YOR, DQ845744; type 9, LAK-1, DQ845742; type 10, RUM, DQ845743. Types 1 and 2 are identical to types of the same number in Bunikis et al. (AY515265 and AY515266, respectively) (6). No sequences matched types 3 and 4 identified by Bunikis and co-workers and are excluded here to avoid confusion. Types 5–10 are newly identified here. ‡DOU detected by PCR in a pool of 3 dead Ornithodoros hermsi. ELD based on spirochetes transmitted by O. hermsi to a laboratory mouse, followed by PCR of infected blood.

Nucleotide sequences were analyzed with Sequencher 4.2 (Gene Codes Corp., Ann Arbor MI, USA). DNA sequences were first aligned with the CLUSTAL V program in the Lasergene software package (DNASTAR Inc., Madison, WI, USA). Alignments were transferred into the MacClade program (*9*) and corrected manually. MacClade output files were opened in PAUP (*10*), and maximum-likelihood neighbor-joining trees were created. Alignments were also created with the DNasp package of algorithms (www.ub.es/dnasp) to calculate mean nucleotide diversity (π) per aligned base. A full heuristic search with 1,000 bootstrap replicates was performed to test the robustness of clade designations.

All stages of colony-reared O. hermsi were fed on hand-held 14-day-old chickens (Gallus domesticus) or 10-day-old northern bobwhite quail (Colinus virginianus) acquired from commercial hatcheries. Ticks were also fed on 5- to 10-day-old mice that were unrestrained in plastic jars with plaster-of-paris bases and screened lids. Nonfeeding ticks were kept at 85% relative humidity, 20°–22°C, natural photoperiod (Hamilton, MT), and observed for development in their life cycle.

B. hermsii DAH and REN were tested first for infectivity in mice as described (*4*). Next, 0.1 mL of blood with ≈5 × 106 spirochetes from each mouse was injected intraperitoneally into four 4-day-old chickens. The inoculum of DAH-infected blood was split between intraperitoneal and subcutaneous sites in 1 bird. The 8 birds were monitored for spirochetemia for 7 days postinoculation by intravenous collection of blood from the wing’s brachial vein and darkfield microscopic examination (×400) of the wet, unstained blood. The Rocky Mountain Laboratories Animal Care and Use Committee approved the tick feeding and experimental inoculations (Protocol nos. 03–31 and 05–17).

## Results

**Figure 2:**
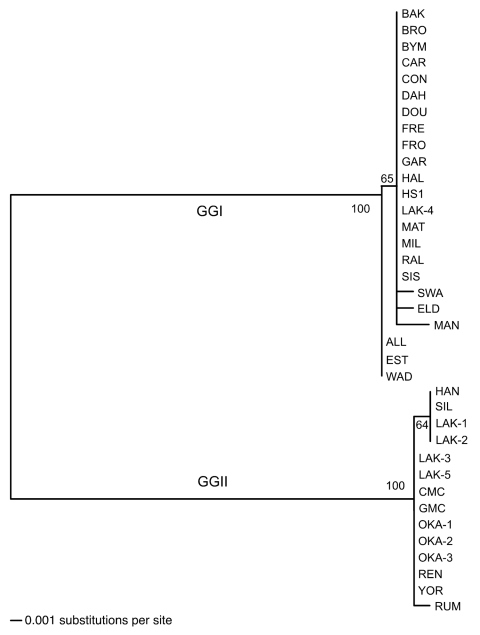
Phylogram of the intergenic spacer sequences of 37 Borrelia hermsii isolates. The tree was constructed with ClustalV and the neighbor-joining method with 1,000 bootstrap replicates. Numbers at the nodes are the percentages of bootstraps that supported this pattern. The scale bar for the branch lengths represents the number of substitutions per site. An unrooted tree is shown because a gap in the alignment with B. turicatae resulted in the removal of a polymorphic site in some GGII isolates of B. hermsii.

The phylogram based on the IGS sequences separated the 37 isolates of B. hermsii into genomic group I (GGI) and genomic group II (GGII) as defined previously (Figure 2) (*4*). Alignment of the sequences identified 3 indels (gaps resulting from insertions or deletions) of 1, 13, and 13 bp between the 2 groups. All GGI isolates contained an IGS sequence of 663 bp compared with 690 bp in all GGII isolates. Aligned sequences for all isolates, excluding the indels, demonstrated that the IGS region was more polymorphic and had greater nucleotide diversity between the 2 genomic groups than did the other loci (Table 2). IGS sequences varied little within each genomic group, with 5 polymorphic sites in GGI and only 2 polymorphic sites in GGII. GGI and GGII contained 5 and 3 sequence types, respectively (Table 1), with only a 1- or 2-bp difference within either group. IGS sequences were determined for 6 isolates of B. parkeri and 8 isolates of B. turicatae described previously (*5*) (GenBank accession nos. DQ855545–DQ855558) but are not discussed further in this study.

**Table 2:** Descriptive statistics for 5 loci in Borrelia hermsii GGI and GGII, western North America*

Group	Locus†	Samples‡	Alleles	Bp	Indels§	Polymorphisms (%)	π¶
All isolates	IGS	37	8	663/690	3	54 (8.1)	0.03648
GGI	IGS	23	5	663	0	5 (0.75)	0.00088
GGII	IGS	14	3	690	0	2 (0.29)	0.00084
All isolates	16S rRNA	35	2	1,273	0	5 (0.39)	0.00194
GGI	16S rRNA	21	1	1,273	0	0	0
GGII	16S rRNA	14	1	1,273	0	0	0
All isolates	flaB	36	5	1,002	0	16 (1.6)	0.00634
GGI	flaB	22	3	1,002	0	5 (0.5)	0.00150
GGII	flaB	14	2	1,002	0	1 (0.1)	0.00044
All isolates	gyrB	35	5	1,902	0	40 (2.1)	0.00997
GGI	gyrB	21	4	1,902	0	3 (0.16)	0.00046
GGII	gyrB	14	1	1,902	0	0	0
All isolates	glpQ	36	9	1,020/1,026	1	37 (3.6)	0.01721
GGI	glpQ	22	6	1,020	0	4 (0.39)	0.00113
GGII	glpQ	14	3	1,026	0	2 (0.19)	0.00087

*GGI, genomic group I; GGII, genomic group II. †Nucleotide sequences for 16S rRNA, flaB, gyrB, and glpQ for isolates not included previously (4) have been deposited in the GenBank database with accession nos. DQ855527 to DQ855544. ‡The number of samples varies because only the IGS sequence was determined for DOU and only IGS, flaB and glpQ sequences were determined for isolate ELD. §IGS with 3 indels of 1, 13, and 13 bp; glpQ with 1 indel of 6 bp. ¶π, mean nucleotide diversity at each aligned position.

IGS sequences varied in their ability to identify unique genotypes associated with geographic clusters of isolates within each genomic group when compared to other loci. The phylogram based on flaB sequences was nearly identical to the IGS phylogram (data not shown). However, neither the IGS nor flaB sequences separated SIS and RAL from the other GGI isolates. SIS and RAL came from a tick and patient, respectively, from the same cabin in northern California (*11*). IGS and flaB sequences for both isolates were identical to most other GGI isolates. Yet, gyrB and glpQ sequences were identical in these 2 isolates and separated them from all or all but 1 of the other GGI isolates, respectively, from other localities (*4*).

Isolates LAK-1, LAK-2, SIL, and HAN in GGII had identical IGS sequences that were different from all other isolates in their genomic group by 1 base. These isolates originated from patients infected in western Montana (LAK-1 and LAK-2) and northern Idaho (HAN and SIL). The locations of exposure are only ≈110 miles (183 km) apart, which suggests a unique IGS genotype for this geographic cluster of isolates. These 4 isolates also contained a flaB allele unique from all other isolates, and LAK-1, LAK-2 and SIL contained a gyrB allele unique from the other isolates (*4*).

The B. hermsii isolates from 5 patients infected in 2 cabins on Wild Horse Island, Flathead Lake, Montana, USA, in 2002 and 2004 were especially intriguing. Both IGS and multilocus sequence analysis typed LAK-4 in GGI and LAK-1, −2, −3, and −5 in GGII. The 3 patients infected in 2004 slept in the same bed; GGI spirochetes were isolated from 1 patient, and GGII spirochetes were isolated from the others. Among the 4 GGII isolates, LAK-1 and LAK-2 originated from 1 cabin (2002) and had identical IGS, 16S rRNA, flaB, gyrB, and glpQ sequences. LAK-3 and LAK-5 came from the other cabin (2004), and these isolates also had identical sequences for the 5 loci examined. However, LAK-1 and LAK-2 contained IGS, flaB, and glpQ sequences that each varied by 1 base compared to LAK-3 and LAK-5 sequences. The presence of 3 IGS, flaB, and glpQ sequences among the 5 isolates suggests multiple past introductions of B. hermsii to the island.

Another B. hermsii isolate of interest was YOR from California (*12*), which had an identical IGS sequence to most other GGII isolates (Figure 2). Multilocus sequencing also showed YOR was identical to isolates from southern British Columbia and northern Idaho (*4*). Therefore, this isolate originated far from where all other GGII isolates came (Figure 1).

The presence of multiple genotypes of B. hermsii on Wild Horse Island (nearest shoreline is 2 km from the mainland), and the finding of identical genotypes separated over large geographic distances, suggested that birds might play a role in dispersing these spirochetes in nature. For this to occur, birds must be suitable hosts for O. hermsi. Therefore, we attempted to feed various stages of O. hermsi on chickens and quail. Larvae, nymphs, and adults all fed within 10 to 30 minutes on the birds, and the ticks survived with a low proportion of deaths (Table 3). Female ticks laid viable eggs that produced larvae, which survived 7 months until they fed on mice. Larvae that fed on chickens molted to first nymphs, and these ticks fed on quail or mice up to 9 months later. These results demonstrated that these experimental birds were suitable hosts for all stages of O. hermsi.

**Table 3:** Number and stage of Ornithodoros hermsi fed on chickens, quail, or mice*

Cohort A	Cohort B	Cohort C
2 M, 2 F on chicken	310 L on chicken†	184 first N on chicken
127 L on mouse	261 first N on quail 42 first N on mice	179 second N on mouse
124 first N on mouse	84 second N on mice	169 third N on mouse

*M, males; F, females; L, larvae; N, nymphs. †After larvae were fed on chickens, the first nymphs were fed on quail or mice. Some of the resultant second nymphs were used in other experiments, hence the smaller number of second nymphs fed on mice.

Eight 4-day-old chickens were inoculated with B. hermsii and examined for spirochetemia levels for the next 7 days. Only the 1 bird injected subcutaneously had a detectable spirochetemia on day 3 postinoculation, with 2 spirochetes seen in 25 fields, which indicated that birds may be more susceptible to infection by this route of inoculation and also possibly by tick bite.

## Discussion

The IGS region separated all isolates of B. hermsii into GGI or GGII defined by the other 4 loci. The IGS, 16S rRNA, flaB, gyrB, and glpQ sequences each contained unique positions (signature bases) that were conserved among all isolates of each genomic group that are suitable for typing B. hermsii in 1 of the 2 groups (available in GenBank). However, the IGS sequences were more polymorphic between the 2 genomic groups than were the other loci, and the indels in this region created unique sequences in GGII isolates that were absent in GGI isolates. Thus, the IGS region is an efficient target for typing B. hermsii into 1 of the 2 genomic groups. Hovis and co-workers recently sequenced the factor H–binding locus, fhbA, in 24 of our 37 isolates of B. hermsii (*13*). However, possible horizontal transfer of this plasmid-encoded gene between spirochetes makes this gene unsuitable for genomic group typing.

Based on our results, the 9 isolates of B. hermsii that Bunikis et al. typed previously with the IGS sequence all belong to GGI, including the spirochete identified in the northern spotted owl (Strix occidentalis caurina) (*6*,*14*). Their type 2 isolates (AY515266) from New Mexico and Colorado had identical IGS sequences to isolates from Utah (ALL), Colorado (EST-7), and eastern California (WAD). Bunikis et al. also deposited an unpublished IGS sequence for a human isolate of B. hermsii from Idaho (LPO; AY515270), which they did not type but which is identical to the IGS sequence in 9 of our GGII isolates (type 8). Our data, combined with those of Bunikis et al. (*6*), include 46 IGS sequences from B. hermsii isolates or B. hermsii–infected material. B. hermsii YOR is the only GGII isolate known from outside the inland Northwest where all other isolates of this group originated. Dispersal of spirochetes by birds could explain the occurrence of identical genotypes found in distant locations.

IGS sequences varied little within either genomic group, but as with the other loci the sequences were more polymorphic in GGI than in GGII. The 3 indels in this noncoding region could have arisen either by deletions in GGI or insertions in GGII, although the bias may be for deletions, as has been proposed for other bacterial genomes, including B. burgdorferi (*15*). The fewer polymorphisms in all loci examined, the larger IGS sequence, and the restricted geographic distribution of isolates all suggest that GGII is a more recent derivative of GGI.

Spirochetes in both genomic groups of B. hermsii are transmitted by the same species of tick (*4*), sympatric in the northern parts of their range, and pathogenic in humans. Evidence for horizontal transfer of the variable tick protein (vtp) gene between spirochetes in the 2 genomic groups suggests that dual infections have occurred in the same host, most likely ticks (*4*). Therefore, what might have driven the selection for 1 clone (GGII) of B. hermsii to diverge from another (GGI) is intriguing to consider. Might there have been a significant period of time when the populations were isolated from each other? Or might there be different primary enzootic vertebrate hosts that maintain these different spirochetes in nature? Only 1 isolate we examined (EST-7 in GGI) came from an enzootic host, a chipmunk in Colorado (*16*). The other isolates came from ticks or human patients, who are only accidental hosts for the spirochetes, so this tells us nothing about their vertebrate hosts in the wild.

The possible role of birds in the ecology and epidemiology of tickborne relapsing fever caused by *B. hermsii* in western North America is worthy of further investigation. The dogma for 70 years has been that pine squirrels (*Tamiasciurus* spp.) and chipmunks (*Tamias* spp.) are the primary vertebrate hosts of *B. hermsii* and its tick vector *O. hermsi* (*1**,**17**–**19*). During our investigation of the relapsing fever outbreak in Montana, we found *O. hermsi* and dead American robin chicks (*Turdus migratorius*) in nest material from the cabin’s attic (*20*). An outbreak associated with another cabin on the same island in 2004 (source of isolates LAK-3, -4, and -5 in this report) led the infected family to suspect that barn swallows (*Hirundo rustica*) were the hosts of the infected ticks, although no investigation was done.

Few *O. hermsi* have been found in association with birds. In 1949, Gregson collected 26 *O. hermsi* in a bluebird (*Sialia* sp.) nest in southern British Columbia. Five fledgling bluebirds were in the nest, and most ticks had recently fed (*21*). Furman and Loomis reported 9 *O. hermsi* in a house sparrow (*Passer domesticus*) nest and 2 *O. hermsi* in a California gull (*Larus californicus*) nest at Mono Lake, California (*22*). However, the later record is probably accidental as extensive investigation of ticks in the gull colonies on the islands in Mono Lake has never found *O. hermsi*, except where humans slept (*23**,**24*).

*B. hermsii* was identified once in a wild bird, a northern spotted owl found dead in Kittitas County, Washington (*14*). Spirochetes were not isolated, but DNA extracted from the bird’s liver contained 16S rRNA DNA that was 99.6% identical to *B. hermsii* sequences. The spirochete was subsequently called *B. hermsii* on the basis of its IGS sequence, which was compared to those of 8 isolates of this species (*6*). The authors stated that while a nest-associated transmission cycle was possible, the infection more likely resulted from direct transmission from an infected prey animal to the owl.

Little work has explored the susceptibility of birds to infection with relapsing fever spirochetes. *B. duttonii,* a cause of tickborne relapsing fever in Africa, produced a detectable spirochetemia level in chickens (*25*) and grew in chick embryos for 1 month (*26*). The inoculation experiments with *B. hermsii* in chickens suggest that some birds may be suitable hosts for this spirochete in the wild. Further experiments are needed to determine if birds become spirochetemic after being fed upon by infected ticks, and if spirochete densities become high enough in bird blood to facilitate their acquisition back to feeding ticks. Cavity-nesting birds and their young might be ideal hosts for both ticks and spirochetes and should be investigated as possible sources of human infection when established nests are present in tick-infested cabins.

Migratory birds might disseminate *B. hermsii* in nature, as has been found for *B. burgdorferi* and *B. burgdorferi*–infected ticks (*27**–**29*). Immature stages of *Ixodes scapularis* ticks feed for 3 to 6 days and thus can be transmitted long distances by birds. In contrast, all stages of *O. hermsi* are rapid feeders and are unlikely to be carried very far by birds. Nocturnal forest birds such as owls could possibly spread infected ticks or prey short distances while foraging at night. However, we believe that infected birds are more likely to disseminate spirochetes directly than by transporting infected ticks. The information gained from *B. hermsii* isolated from patients has been extremely informative but these spirochetes represent only a small segment of the true populations of these bacteria. More work is needed to acquire additional isolates of *B. hermsii* to determine more fully the true distribution of these spirochetes throughout their entire range and identify the species of enzootic hosts involved in maintaining the spirochetes in nature.
